# Matrix‐assisted laser desorption ionization ‐ mass spectrometry imaging of erlotinib reveals a limited tumor tissue distribution in a non‐small‐cell lung cancer mouse xenograft model

**DOI:** 10.1002/ctm2.481

**Published:** 2021-07-08

**Authors:** Tae Young Kim, Boram Lee, Yonghyo Kim, Yutaka Sugihara, Melinda Rezeli, A. Marcell Szasz, Balazs Dome, Gyorgy Marko‐Varga, Ho Jeong Kwon

**Affiliations:** ^1^ Chemical Genomics Global Research Lab Department of Biotechnology College of Life Science and Biotechnology Yonsei University Seoul Korea; ^2^ Division of Clinical Protein Science and Imaging Department of Clinical Sciences (Lund) and Department of Biomedical Engineering Lund University Lund Sweden; ^3^ Department of Tumor Biology National Korányi Institute of Pulmonology Budapest Hungary; ^4^ Division of Thoracic Surgery Department of Surgery Medical University of Vienna Vienna Austria

Dear Editor,

Erlotinib has been used to treat patients with EGFR‐mutated non‐small‐cell lung cancer (NSCLC) for almost two decades; however, acquired resistance sooner or later develops against its blockade, thus, low efficacy is inevitable in some patients.^1^, ^2^ Many studies have aimed to discern the cause of this resistance by exploring the underlying molecular mechanisms of erlotinib. To investigate an underlying mechanism of erlotinib's resistance, its distribution in tumor, liver, and kidney tissues were analyzed with matrix‐assisted laser desorption ionization mass spectrometry imaging (MALDI‐MSI) in drug‐resistant and drug‐sensitive NSCLC mouse xenograft models. The low *in vivo* distribution of erlotinib in tumor tissues in a drug‐resistant NSCLC mouse xenograft model suggests the existence of a new resistance mechanism in NSCLC.

To investigate the resistance of NSCLC cell lines to erlotinib treatment, the drug effects on proliferation were studied in two NSCLC cell lines, H1299 (EGFR WT; erlotinib‐resistant) and PC9 (Exon19 del; erlotinib‐sensitive).^3^, ^4^ Erlotinib weakly inhibited cell proliferation in the H1299 cells (IC_50_ = 65 μM, Figure 1A). In contrast, robust inhibition was observed in the PC9 cells (IC_50_ = 0.7 μM) (Figure 1B). Additionally, we investigated whether erlotinib inhibited EGFR kinase activity in H1299 cells to confirm previously reported results.^5^ Erlotinib (30 μM) was applied to EGF‐induced H1299 cells to examine the drug effects on the EGFR signaling pathway. As presented in Figure 1C, EGF activated the EGFR, whereafter the activation was suppressed by erlotinib treatment, which led to the suppression of both AKT and ERK phosphorylation. We also used the DARTS assay to investigate the binding of erlotinib to EGFR (Figure 1D).^6^, ^7^ Pronase treatment, that is, digestion, significantly reduced EGFR level. However, this digestion was suppressed by pre‐treatment with erlotinib (30 μM) due to a conformational change induced by erlotinib binding to EGFR. In contrast, the amount of VDAC1, a non‐erlotinib binding protein used as a control, was significantly decreased even when incubated with erlotinib prior to pronase treatment. This implies that erlotinib is directly binding to EGFR to inhibit EGF‐induced EGFR kinase activity. Based on these results, H1299 was selected to explore the erlotinib resistance mechanisms in this study.

**FIGURE 1:**
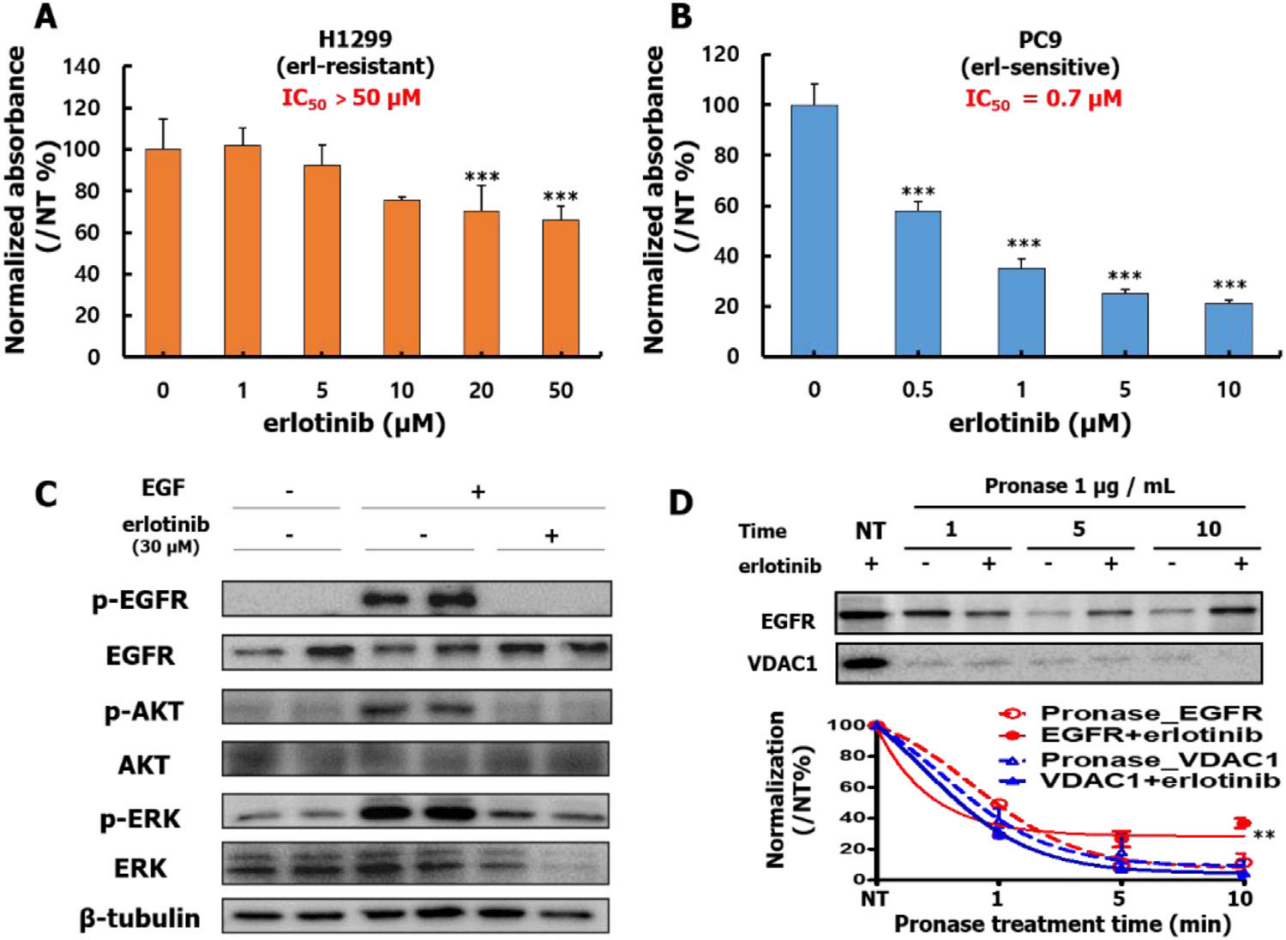
Erlotinib treatment of NSCLC cell lines. (A) Effect of erlotinib on the proliferation of H1299 and (B) PC9 cell lines at 72 hours. IC_50_ values were calculated from the inhibition ratio. The erlotinib IC_50_ in H1299 cells was predicted to be 65 μM. NT indicates dimethyl sulfoxide (DMSO) alone, and it was also used to normalize data of erlotinib treated group. (C) H1299 cells were treated with 30 μM erlotinib for 2 h. After serum starving, the cells were stimulated by adding 100 ng/ml EGF for 15 min. (D) DARTS analysis to determine erlotinib binding with EGFR. DARTS analysis was performed to analyze the interaction of erlotinib to EGFR and VDAC1, an erlotinib non‐binding protein control. All data are the mean ± S.E.M. and CV < 20 % from ≥ 3 independent experiments, ***p < 0.001 vs NT, **p < 0.01 versus Pronase_EGFR, paired t test by GraphPad Prism

Next, the *in vivo* responses of erlotinib in H1299 (erlotinib‐resistant) and HCC827 (erlotinib sensitive) cells were investigated in xenograft mice models. The PC9 cell line was not used in this study because PC9 derived tumors were associated with severe ulceration, which would also be difficult to analyze for MALDI‐MSI.^8^ Additionally, HCC827 cell line harbors exon19 del of EGFR such as PC9 cell line.

Notably, erlotinib did not reduce the volume of H1299 tumors (Figure 2A) in contrast to HCC827 tumors (Figure 3A), which confirmed the cells were erlotinib‐sensitive (IC_50_ = 0.2 μM) (Figure S2), and there was no apparent toxic indication of the erlotinib treatment (Figures 2B and 3B). H1299 tumor tissue, liver, and kidney were isolated from the xenograft mice previously treated with either vehicle (*N* = 4) or erlotinib (*N* = 5) (10 mg/kg) for analyzing erlotinib distribution. The distribution of erlotinib was then examined in the isolated tissue sections with MALDI‐MSI. The erlotinib target protein, EGFR, was visualized with immunofluorescence. The images generated from the vehicle and erlotinib‐treated groups were subsequently compared and are presented in Figures 2C‐2R and 3C‐3R. We confirmed that erlotinib co‐localized with EGFR in the erlotinib‐treated tumor tissue samples.

**FIGURE 2:**
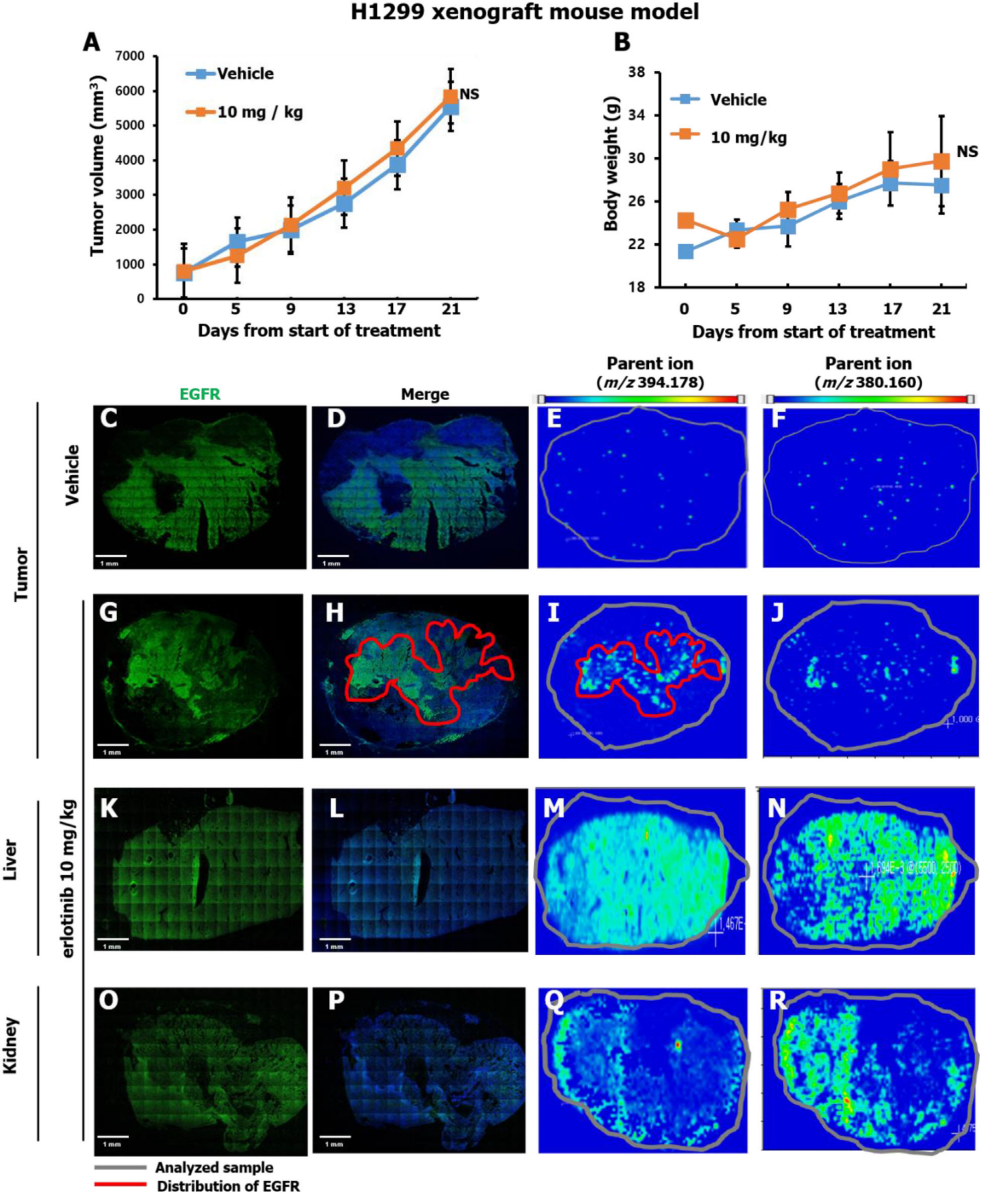
Erlotinib‐resistant mouse xenograft model with H1299 cells and erlotinib distribution in tumor, liver, and kidney tissue. (A and B) Mean tumor volumes ± S.D. and body weight over time in response to treatment with erlotinib are shown. (C‐R) Distribution of erlotinib and its target protein, EGFR, in various tissues from vehicle and erlotinib‐treated mice visualized with MALDI‐MSI and IF, respectively. The nucleus is visualized with Hoechst 33342 (blue) (Figures S12A‐S12D), and EGFR is visualized with IF (green) in the merged images. The gray and the red lines in the MSI images represent boundaries of the analyzed tissue sample and EGFR distributions, respectively. MALDI‐MSI images of metabolites M13/M14 (*m/z* 380.160) and erlotinib (*m/z* 394.178) are presented. These specific *m/z* values of erlotinib were examined by MALDI‐MS using erlotinib standard solution (Figure S1). The signal intensity of specific *m/z* values is presented as an RGB color gradient from blue (low) to red (high). The xenograft mice were treated with either vehicle (*N* = 4) or erlotinib (*N* = 5) (10 mg/kg) for analyzing erlotinib distribution. Abbreviation: NS, not significant.

**FIGURE 3:**
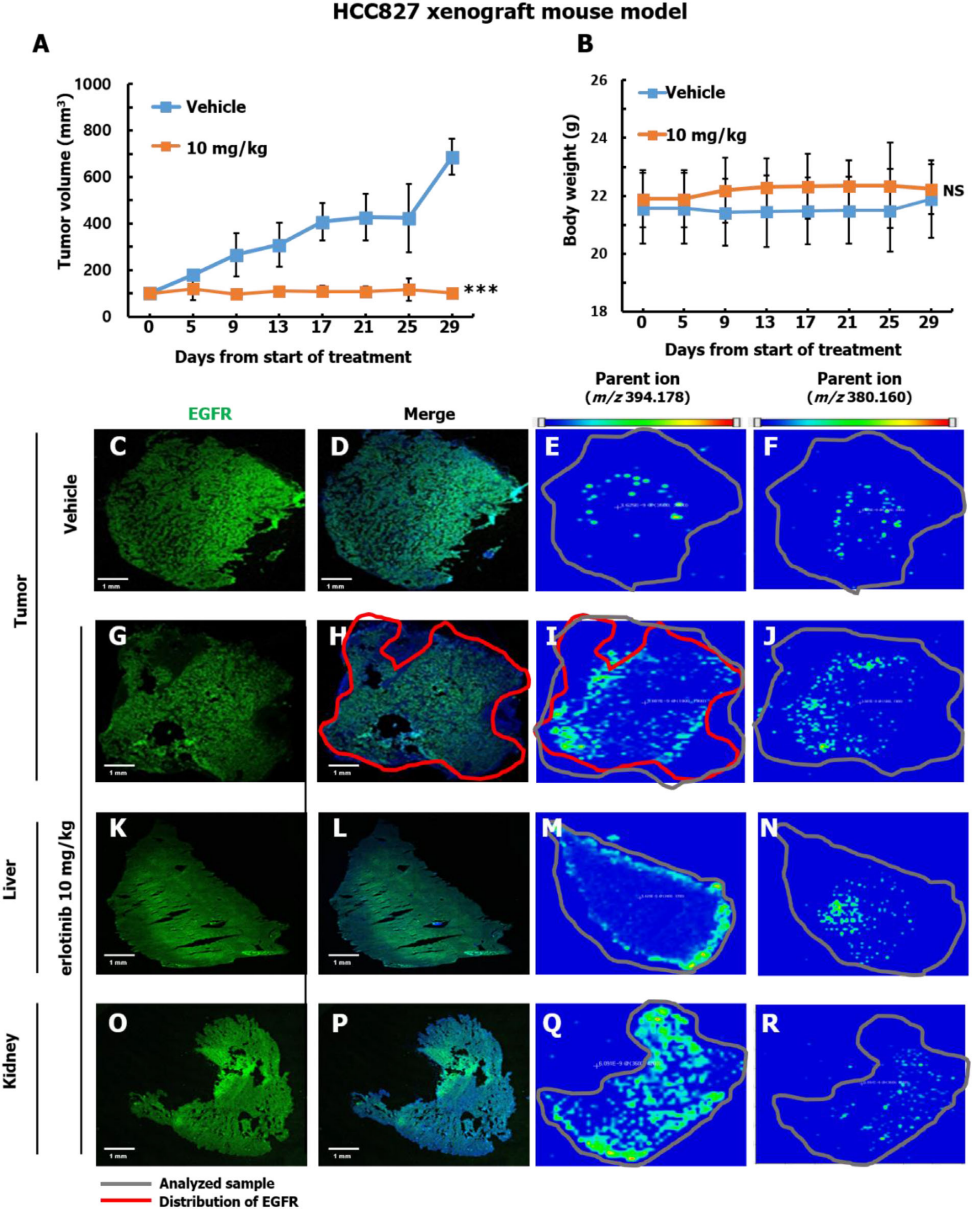
Erlotinib‐sensitive mouse xenograft model with HCC827 cells and erlotinib distribution in tumor, liver, and kidney tissue. (A and B) Mean tumor volumes ± S.D. and body weight over time in response to treatment with erlotinib are shown. ***p < 0.001. (C‐R) Distribution of erlotinib and its target protein, EGFR, in various tissues from vehicle and erlotinib‐treated mice visualized with MALDI‐MSI and IF, respectively. The nucleus is visualized with Hoechst 33342 (blue) (Figures S12A‐S12D) and EGFR is visualized with IF (green) in the merged images. The gray and the red lines in the MSI images represent boundaries of the analyzed tissue sample and EGFR distributions, respectively. MALDI‐MSI images of metabolites M13/M14 (*m/z* 380.160 ) and erlotinib (*m/z* 394.178 ) are presented. These specific *m/z* values of erlotinib were examined by MALDI‐MS (Figure S1) using erlotinib standard solution. The signal intensity of specific *m/z* values are presented as an RGB color gradient from blue (low) to red (high). The xenograft mice were treated with either vehicle (*N* = 6) or erlotinib (*N* = 6) (10 mg/kg) for analyzing erlotinib distribution. Abbreviation: NS, not significant.

In the tumor tissues from H1299 xenograft mouse model, the Total Ion Current (TIC) normalized average signal intensities of erlotinib, and M13/M14 precursor ions (at *m/z* 394.178 and 380.160, respectively) were analyzed in vehicle‐ and erlotinib‐treated mice (Figures 2E, 2F, 2I, and 2J). M13/M14, which are the biologically active metabolites of erlotinib were also detected in erlotinib treated mice tissues.^9^ Our measurement also confirmed that erlotinib signal is detectable in liver and kidneys of drug‐treated mice, showing relatively high signal intensities (Figures 2M, 2N, 2Q, and 2R).

In the tumor tissues from HCC827 xenograft model, the TIC normalized average signal intensities of erlotinib and M13/M14 precursor ions (at *m/z* 394.178 and 380.160, respectively) were analyzed in both vehicle and erlotinib‐treated mice (Figures 3E, 3F, 3I, and 3J). We also confirmed the intensity of the precursor ions in liver and kidney from erlotinib‐treated mice (Figures 3M, 3N, 3Q, and 3R).

These localization data in both xenograft mouse models clearly demonstrate that erlotinib has affected the tumor by binding to EGFR *in vivo*. The average drug signal intensities per tissue unit were calculated for each tissue sample from the HCC827 and H1299 xenograft models to compare the drug distributions in the two *in vivo* mouse groups. We observed that the vehicle‐treated groups exhibited different basic tumor tissue intensities in each model (Figure S3). The average intensity was 4.50E‐8 for HCC827 tumors, 1.36E‐7 for H1299 tumors (Figures S4 and S7), 4.03E‐7 for the liver (Figures S5 and S8), and 1.32E‐8 for the kidney (Figures S6 and S9) in the vehicle‐treated mice. Erlotinib was detected in several tissues but with different intensities in the HCC827 xenograft model group (tumor tissue (13.89), liver (12.32), and kidney (46.42)) similar to the H1299 xenograft model group, where erlotinib was also detected in all three tissue types (tumor tissue (4.55), liver (37.58), and kidney (300.25)) (Figure 4A). Interestingly, as predicted by the high affinity of mutated EGFR in HCC827 cells,^10^ erlotinib content was 2.95 times higher in HCC827 tumors when compared to H1299 tumors. Furthermore, erlotinib showed stronger localization in the kidney of the drug‐treated H1299 xenografts. It is noteworthy that erlotinib was highly localized in normal organ tissues (liver, kidney) in the erlotinib‐resistant H1299 mouse xenograft model.

**FIGURE 4:**
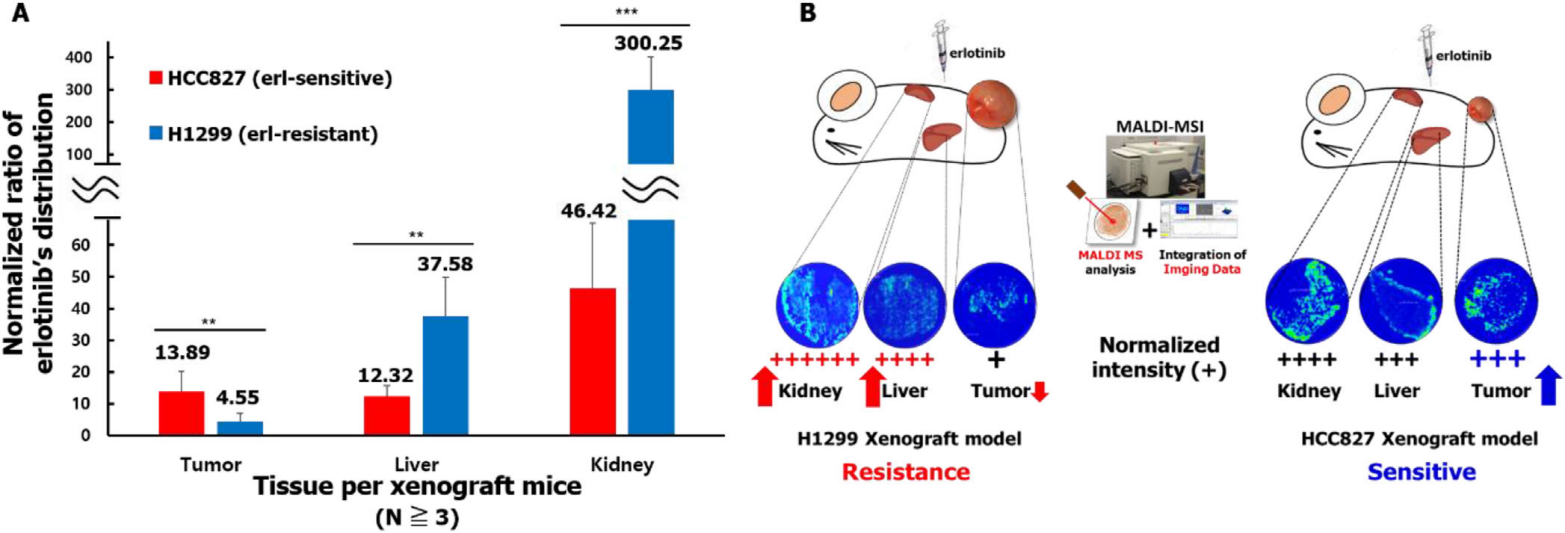
Comparison of normalized erlotinib distributions and schematic summary of this study. (A) Relative erlotinib distribution values (at *m/z* 394.178 ) in HCC827 and H1299 mouse xenografts. All values were calculated from MALDI‐MSI data in triplicate and mean values ± S.D are presented, ***p < 0.001 and **p < 0.01 when compared to control and analyzed with Student's *t*‐tests. (B) Mouse xenograft models were constructed with erlotinib‐resistant (H1299) or erlotinib‐sensitive (HCC827) NSCLC cell lines, and both mouse groups were treated with erlotinib. MALDI‐MSI was used to visualize the distribution of erlotinib in the tissues. For the comparison of erlotinib sensitive and resistant tumor models, the erlotinib signal in each tissue was quantified and normalized with tissue size. Erlotinib had a higher overall signal intensity in tumors from the HCC827 mouse model when compared to tumors from the H1299 mouse model. In addition, erlotinib was highly distributed in normal liver and kidney tissues in the H1299 mouse model. These data provide a new mechanism for *in vivo* erlotinib resistance

In summary, these results demonstrate that in erlotinib‐resistant H1299 xenografts, erlotinib preferentially distributed in the liver and kidney rather than in the tumor tissues (Figure 4B). Although erlotinib still has a binding affinity to the target protein, EGFR, even in the H1299 tumor cells, the reduced distribution within in tumor tissues suggests a new mechanism of erlotinib resistance *in vivo*.

## CONFLICT OF INTEREST

The authors declare that there is no conflict of interest.

## FUNDING INFORMATION

This work was partly supported by grants from the National Research Foundation of Korea and was funded by the government of the Republic of Korea (MSIP; 2015K1A1A2028365, 2016K2A9A1A03904900), Brain Korea 21 Plus Project, and ICONS (Institute of Convergence Science), Yonsei University, Republic of Korea as well as the Berta Kamprad Foundation, Lund, Sweden and the KNN121510 grant by the National Research, Development and Innovation Office of Hungary.

## AUTHOR CONTRIBUTIONS

Tae Young Kim and Ho Jeong Kwon participated in project conception and experimental design. Tae Young Kim performed cell and molecular biology assays, DARTS assays, in vivo assays (mouse xenograft models injecting H1299 and HCC827), and analyzed the data. Tae Young Kim, Boram Lee, Melinda Rezeli, Yonghyo Kim, and Yutaka Sugihara analyzed the MALDI‐MSI data. Tae Young Kim, A. Marcell Szasz, Balazs Dome, Melinda Rezeli, Gyorgy Marko‐Varga, and Ho Jeong Kwon wrote the paper. All authors edited and approved the final manuscript.

## DATA AVAILABILITY STATEMENT

Materials are available upon a reasonable request from the corresponding author.

## Supporting information

Supporitng information
