# Supplementary material for: Matrix‐assisted laser desorption ionization ‐ mass spectrometry imaging of erlotinib reveals a limited tumor tissue distribution in a non‐small‐cell lung cancer mouse xenograft model
Source: Clin Transl Med. 2021 Jul 8;11(7):e481. doi: 10.1002/ctm2.481 (PMC8265169; doi:10.1002/ctm2.481)
Supplement: Supplementary file 1 — Supporitng information [file CTM2-11-e481-s001.docx]

Matrix assisted laser desorption ionization - mass spectrometry imaging of erlotinib reveals a limited tumor tissue distribution in a non-small-cell lung cancer mouse xenograft model

**MATERIALS AND METHODS**

Materials

Erlotinib (N-[3-ethynyl phenyl]-6, 7-bis [2-methoxyethoxy]-4-quinazolinamine, C_22_H_23_N_3_O_4_, ≥ 98 %) was purchased from Cayman Chemical (Ann Arbor, MI). Trifluoroacetic acid (TFA), methanol (MeOH) and α-cyano-4-hydroxycinnamic acid (CHCA) were purchased from Sigma-Aldrich (St Louis, MO). Acetonitrile (ACN) and acetic acid (hyper-grade for LC-MS) were purchased from Merck (Darmstadt, Germany). Anti-EGFR (#4267, 1:3000) and anti-phospho-EGFR (#2236, 1:3000) antibodies were purchased from Cell Signaling (Beverly, MA). Anti-β-tubulin (ab 15568, 1:3000) antibody was purchased from Abcam (Cambridge, UK). Matrigel (Cat. No. 354234) was purchased from Corning (Corning, NY).

Cell culture

Dr. Ho-Young Lee (College of Pharmacy, Seoul National University) kindly provided the human NSCLC cell lines (PC9, HCC827, and H1299). The cell lines were maintained in RPMI 1640 medium that was supplemented with 10% fetal bovine serum (Life Technologies, Gaithersburg, MD). All cell lines were maintained at 37°C in a humidified incubator with 5% CO_2_ and a media pH of 7.4.

Proliferation assay

Cell proliferation was measured with 3-(4,5-dimethylthiazol-2-yl)-2,5-diphenyltetrazolium bromide kits (MTT, VWR Life Science, Radnor, PA). Briefly, HCC827, PC9, and H1299 cells were seeded in 96-well plates at densities of 3 × 10^3^, 3 × 10^3^, and 2 × 10^3^ cells/well, respectively. The cells were then incubated overnight and treated with the compound (erlotinib) for 24 to 72 hours. Then, 25 μL of MTT (2 mg/mL) in phosphate-buffered saline (PBS) were added for 3 hours. Cell proliferation was quantified by measuring absorbance at 595 nm (MTT) on a Victor 5 multi-label plate reader (Perkin Elmer, Waltham, MA).

DARTS assay

Drug affinity responsive target stability (DARTS) is a label-free method; therefore, it does not require any chemical modification of the compound. The method is based on the concept that ligand-bound proteins exhibit enhanced stability against proteolysis when compared to ligand-unbound proteins. Indeed, a target protein bound to its ligand exhibits increased or decreased stability upon protease treatment, which can be detected by an altered protein band intensity during SDS-PAGE when compared to the ligand-free protein.[^1^](#_ENREF_1)^,^ [^2^](#_ENREF_2)

To acquire a proteome pool in this study, H1299 cells were scraped and lysed with the Mem-PER Plus membrane protein extraction kit (Cat. No. 89842, Thermo Fisher Scientific). Membrane proteins were prepared according to the manufacturer’s instructions. After measuring protein levels with the bicinchoninic acid (BCA) assay, the samples were diluted to a protein concentration of 1 mg/mL. To saturate the drug-protein binding interaction, samples were incubated with a high concentration (1 mM) of erlotinib at 4°C for 90 minutes. Samples were then treated with Pronase (Roche) at 25°C to induce proteolysis and were analyzed with immunoblotting.

Immunoblot analysis

Cell lysates were separated with 8, 10, or 12.5 % SDS-PAGE and transferred to polyvinylidene fluoride membranes (Millipore, Billerica, MA) using standard electroblotting procedures and the Trans-Blot SD Semi-Dry transfer system (Bio-Rad, Hercules, CA). Blots were blocked and immunolabeled overnight at 4°C with primary antibodies. Immunolabeling was detected with an enhanced chemiluminescence kit (Bio-Rad) according to the manufacturer’s instructions. Images were quantified with Image Lab™ software (Bio-Rad) with β-actin as an internal control.

*In vivo* tumor model and treatment

The mice were housed in the Specific Pathogen-Free facility in the Laboratory Animal Research Center at Yonsei University. All mice were handled according to the Institutional Animal Care and Use Committee (IACUC-A-201702-139-01) and international guidelines on the ethical use of animals. Cell suspensions containing 5×10^6^ H1299 or HCC827 cells in 100 μL of 1× PBS/Matrigel (1:1) were subcutaneously implanted into the dorsal flank of athymic nude mice (4 week old female Balb C nu/nu, Orient Bio, Seoul, South Korea). After 2 weeks, the mice were randomly divided into two groups and intraperitoneally (i.p.) treated with vehicle or erlotinib (10 mg/kg) for 21 days (4 days per week). The vehicle treatment was ethanol (2%), Tween80 (15%), and filtered saline (83%). The erlotinib was dissolved in the vehicle solution at 10 mg/kg. All animals at all-time points were sacrificed 15 minutes after the last drug treatment. The tumor volume and mouse body weight were measured daily using the following formula: π/6 × length × width × height. The tumors were surgically removed and gradually frozen over a 2-minute period in a plastic container that was floating on a bath of isopentane and dry ice (−70°C).

Matrix deposition

To image the drug in the tissues, 10 μm frozen sections were cut on a cryotome (Leica CM 1950) and placed on a glass slide (Superfrost™ Plus, Thermo Fisher Scientific). A TM-sprayer™ was used to deposit matrix solution (α-CHCA) on the tissue sections. The α-CHCA concentration was 5 mg/mL in 50 % MeOH, 0.1 % TFA and 5 mL of the matrix solution was applied to each tissue sample. To achieve a relatively dry spray, a block temperature of 35 °C was selected. The spraying pattern was a homogenous thin film with a drying time of 30 seconds. The matrix flow rate through the pump was 100 μL/minute and the air pressure was 10 psi.

Erlotinib detection and drug distribution analysis

A MALDI LTQ Orbitrap XL mass spectrometer (Thermo Fisher Scientific, Bremen, Germany) was used to characterize the drugs on a MALDI target plate and to detect the erlotinib signal in tissue samples. For MSI analysis, the full mass spectra were obtained from the Orbitrap mass analyzer at a resolution of 60,000 (at *m/z* 400). Tissue sections were sampled in positive-ion mode using a *m/z* range of 140 − 800 and a 100 μm raster size. The nitrogen laser was applied at 10.0 μJ with AGC OFF. For MS/MS data collection, erlotinib (*m/z* 394.17) was fragmented by collision-induced dissociation with an isolation window of *m/z* 2.0, using 60 % NCE (normalized collision energy), 30 ms activation time, and an activation Q of 0.250. The minimal signal required for MS/MS spectra generation in the linear ion trap was 500 counts. The spectra were acquired with Xcalibur™ v 2.1, and visualization of the drug and fragment ions was performed with ImageQuest™ v 1.0.3 (Thermo Fisher Scientific, San José, CA).

Statistical analysis

All results are presented as the mean ± standard error of the mean (±S.E.M) and all statistical analyses were performed with GraphPad Prism (v. 5.00 for Windows, GraphPad Software, San Diego, CA, www.graphpad.com). The average drug signal intensities of the vehicle and, erlotinib treated groups were measured with the "average spectra within a selected area" function in ImageQuest (v.1.0.3 build 132, ImageQuest Software, San José, CA). The average drug signal intensity was divided by the tissue size for each sample, and these normalized values (CV < 20 %, each tissue group in triplicate) were used to compare the control and test groups. Student’s t-tests were applied to determine the statistical significance between the control and test groups. A p-value < 0.05 was considered statistically significant.

**Discussion**

Despite extensive studies on the molecular and cellular mechanisms of EGFR kinase inhibitor resistance, the issue remains unsolved in NSCLC and must be addressed to improve chemotherapeutic efficacy, thereby extending the patient end-point. We performed MALDI-MSI combined with IF for investigation of EGFR expression and the spatial distribution of the EGFR inhibitor, erlotinib, in H1299 and HCC827 mouse xenograft models. Erlotinib was detected in the drug-treated tumor tissues and the drug distribution positively correlated with the target protein, proven by image overlay of EGFR-IF and erlotinib localization by MSI (Figure 2D, 2I and Figure 3D, 3I).

Erlotinib is an effective agent in patients with metastatic NSCLC that harbors EGFR activating mutations, but it is ineffective against EGFR wild type tumors.[^3^](#_ENREF_3) These efficacy differences are due to increased erlotinib affinity in specific EGFR mutants, namely Exon19 del[^4^](#_ENREF_4) and L858R[^5^](#_ENREF_5), when compared to wild type EGFR. Consistent with previous reports, we confirmed a higher distribution of erlotinib in the tumor tissue (13.89) from HCC827 (Exon19 del EGFR) xenografts when compared to the tumor tissue (4.71) from H1299 (wild type EGFR) xenografts. Our study presents EGFR status based on a MALDI-MSI study data set. Additional studies with MALDI-MSI to analyze the distribution of other EGFR inhibitors, including apatinib and gefitinib, and to compare their binding affinities on a molecular level are on-going.

Interestingly, erlotinib treatment decreased EGFR expression in H1299 (wild type EGFR) tumors but had no effect in HCC827 (Exon19 del EGFR) tumor cells. A previous study have reported that decreased EGFR expression was due to EGFR endocytic degradation, which also inactivated the EGFR signaling pathway.[^6^](#_ENREF_6) Degradation was dependent on the EGFR subtype (wild type, Exon19 del, or T790M/L858R) and was internalized the quickest in wild type EGFR. Consistent with these previous results, we observed a higher degree of EGFR was degradation in H1299 tumor tissue as compared to the HCC827 tumor tissue (Figure 2D, 2I and Figure 3D, 3I). It is possible that decreased EGFR expression may contribute to the increased erlotinib distribution to normal tissues (liver and kidney) and contributed to the poor erlotinib efficacy within the H1299 tumors. Accordingly, the efficacy of erlotinib in wild type EGFR NSCLC tumors could be increased by inhibiting EGFR degradation with a dynamin inhibitor.[^6^](#_ENREF_6)^,^ [^7^](#_ENREF_7) Similarly, it has been reported that maintaining surface EGFR by suppressing degradation leads to enhanced erlotinib efficacy and transforms tumors from a resistant to a sensitive status.[^8^](#_ENREF_8) Based on this report, it is important to highlight the data presented in Figure 2D, 2I and Figure 3D, 3I, which demonstrate that erlotinib promoted EGFR degradation *in vivo*. EGFR internalization, which is known as the primary mechanism of EGFR degradation, needs a ligand (such as EGF) to induce a conformational change that promotes internalization. Notably, the ligand can be presented in extremely low, pico-molar concentrations.[^9^](#_ENREF_9) This suggests that erlotinib is a major factor that contributes to EGFR degradation due to conformational changes that occur upon ligand (erlotinib) binding. Additionally, it supports the hypothesis that the altered structure of EGFR after erlotinib binding could enhance the downstream affinity to trafficking proteins such as; SOC5[^8^](#_ENREF_8) and Sortilin,[^10^](#_ENREF_10) respectively, which are used for internalization.

The ATP-binding cassette (ABC) transporter confers resistance to several classes of cancer chemotherapeutic agents and tyrosine kinase inhibitors.[^11^](#_ENREF_11) It is a membrane protein that transports a variety of structurally unrelated substrates out of cells in an energy-dependent manner.[^12^](#_ENREF_12) However, neither the H1299 nor the HCC827 cell line exhibits ABC transporter activity that is indicative of drug resistance. This has been validated by the accumulation of Hoechst 33342 within H1299 and HCC827, which is an ABC transporter substrate.[^11^](#_ENREF_11) Although this specific study did not focus on the H1299 and HCC827 cell lines, the high expression and activity of the ABC transporter in other NSCLC cell lines, and its role in erlotinib drug resistance remain to be uncovered.

Despite the dramatic efficacy of erlotinib for initial tumor treatment, it is widely known that the drug leads to resistance and subsequently, low efficacy in both xenograft models and in the clinic.[^13^](#_ENREF_13)^,^ [^14^](#_ENREF_14) Recent molecular studies on *in vivo* tumor growth have revealed that EGFR DNA mutations contributes to the reduced erlotinib efficacy.[^15^](#_ENREF_15) Despite these advancements, additional research is imperative to understand the processes involved in the development of erlotinib resistance. For this reason, the non-specific distribution of erlotinib to other tissues, including the liver and kidneys, has the potential to provide new insights that can improve efficacy of erlotinib.

**Supplementary Figures**


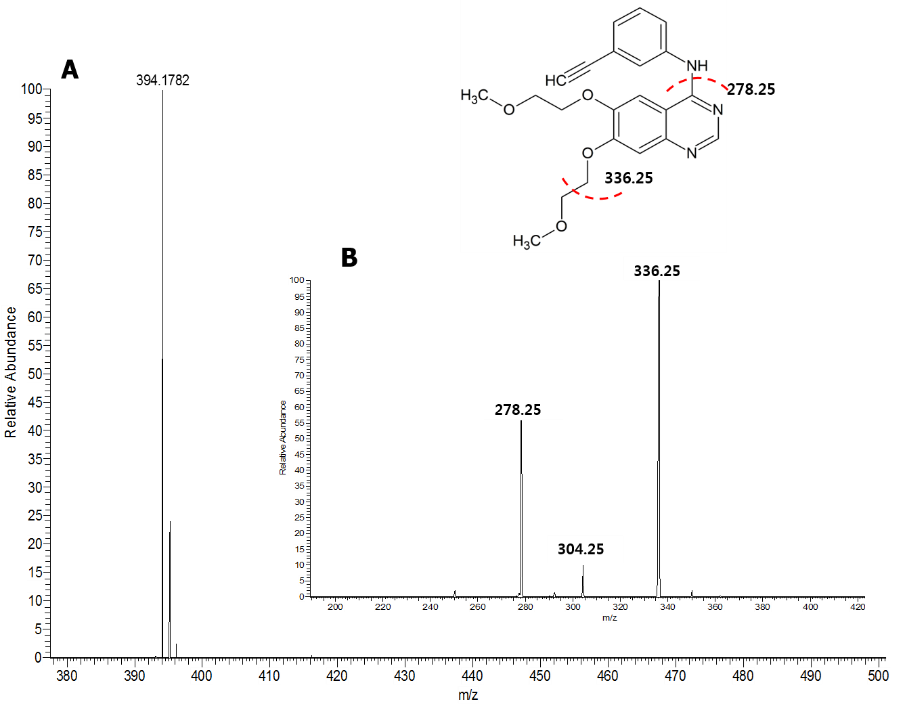


**Figure S1. Ionization of erlotinib with α-CHCA.** (A) Full mass spectrum of erlotinib (*m/z* 394.1782). (B) MS/MS spectrum from a tandem mass spectrum of erlotinib isolating the *m/z* 394.1782 and CID fragmented in the linear ion trap mass analyzer with chemical structure and fragmentation properties.


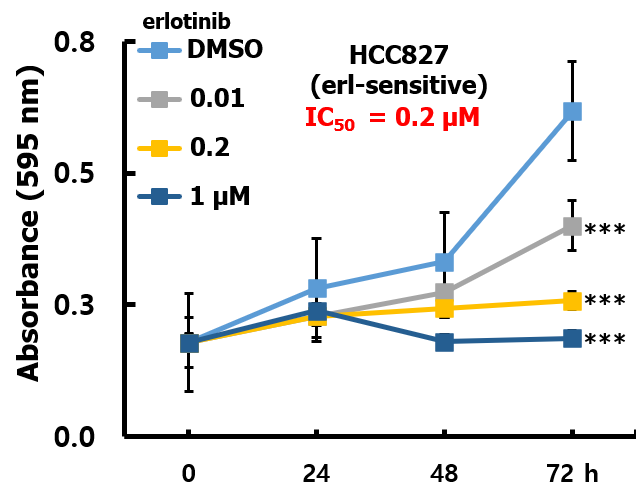


**Figure S2. Proliferative effect of erlotinib in HCC827.** Anti-proliferative effect of erlotinib on erlotinib-sensitive (HCC827) cell line. It was measured by MTT assay after treatment of erlotinib for 72 h. All data points are the mean ± S.E.M. from ≥ 3 independent experiments performed in triplicate. *** *p* < 0.001 vs control.
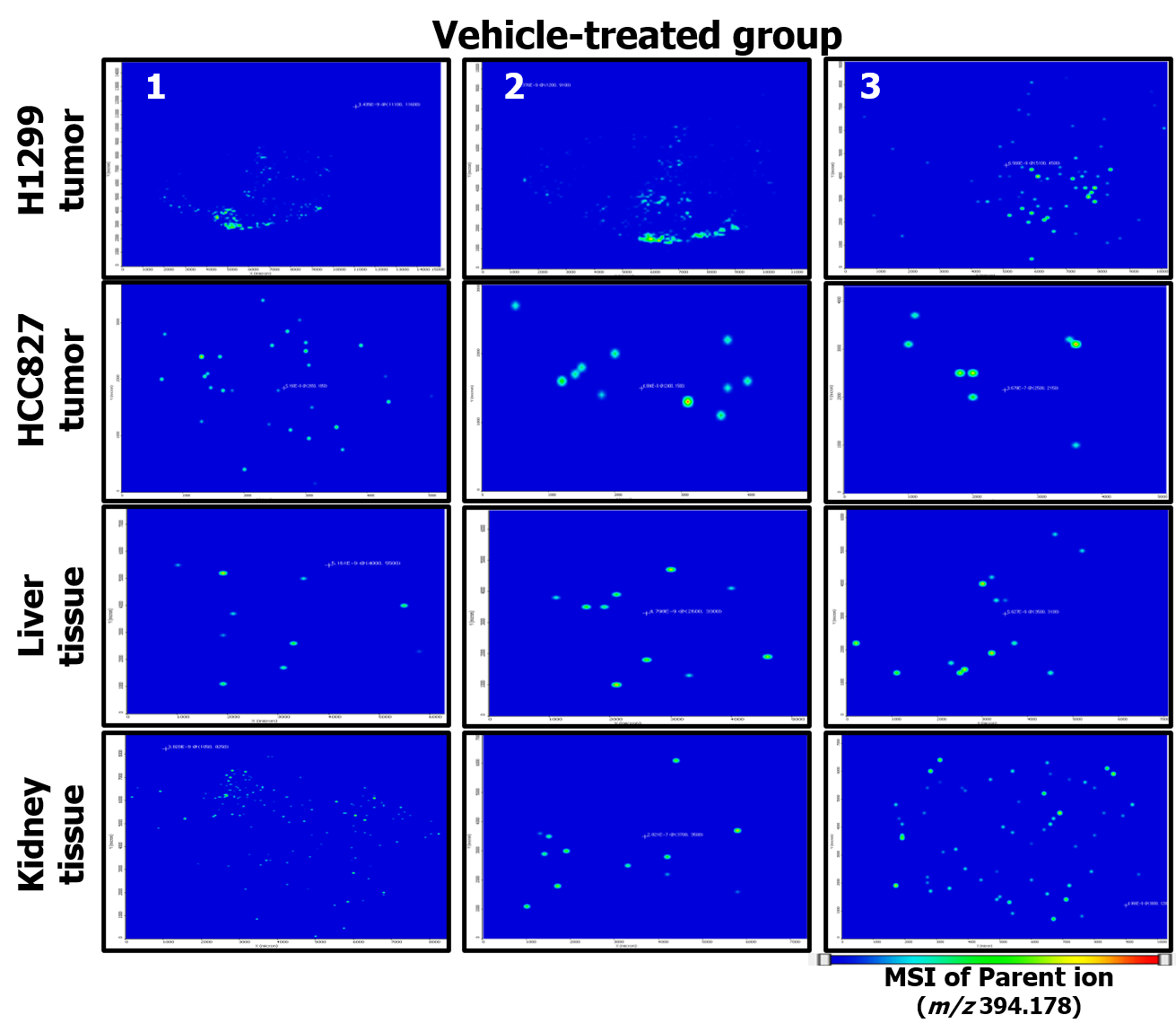
**Figure S3. MALDI-MSI data from vehicle treated mouse xenograft models.** Basal distribution levels of erlotinib in tumor, liver, kidney tissues from vehicle-treated mouse xenografts were analyzed with MALDI-MSI. The images show the signal intensity distribution of *m/z* 394.178. The intensity of the selected *m/z* value is shown as an RGB gradient from blue (low) to red (high).


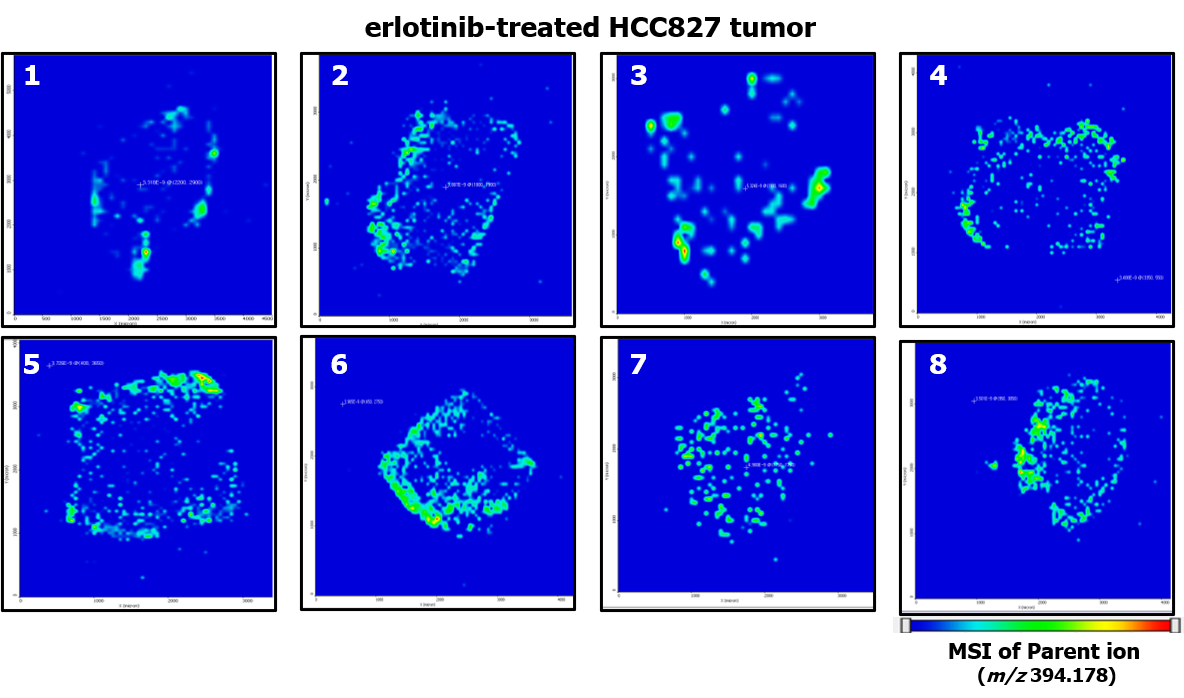


**Figure S4.** **The MALDI-MSI data of drug-treated tumor tissue from HCC827 xenograft mouse model.** Distribution of erlotinib in tumor tissue by MALDI-MSI in HCC827 xenograft mouse. The images show the distribution of the erlotinib signal (*m/z* 394.178). Signal intensity distributions of the specific *m/z* values are shown as an RGB gradient from blue (low) to red (high). Each image represents a tumor sample from different xenograft mouse.


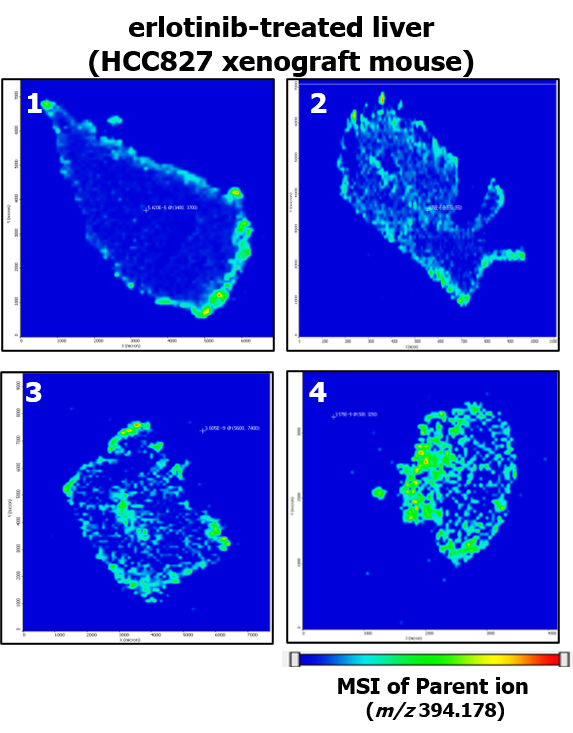


**Figure S5. The MALDI-MSI data of drug-treated liver tissue from HCC827 xenograft mouse model.** Distribution of erlotinib in drug-treated liver tissue by MALDI-MSI from HCC827 xenograft mouse. The images show the distribution of the erlotinib signal (*m/z* 394.178). Signal intensity distributions of the specific *m/z* values are shown as an RGB gradient from blue (low) to red (high). Each image represents a tumor sample from different xenograft mouse.


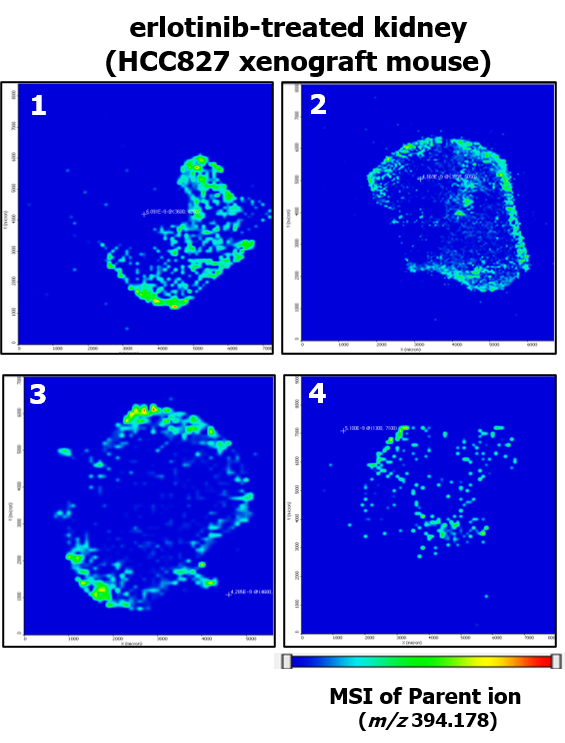


**Figure S6. The MALDI-MSI data of drug-treated kidney tissue from HCC827 xenograft mouse model.** Distribution of erlotinib in drug-treated kidney tissue by MALDI-MSI from HCC827 xenograft mouse. The images show the distribution of the erlotinib signal (*m/z* 394.178). Signal intensity distributions of the specific *m/z* values are shown as an RGB gradient from blue (low) to red (high). Each image represents a tumor sample from different xenograft mouse.


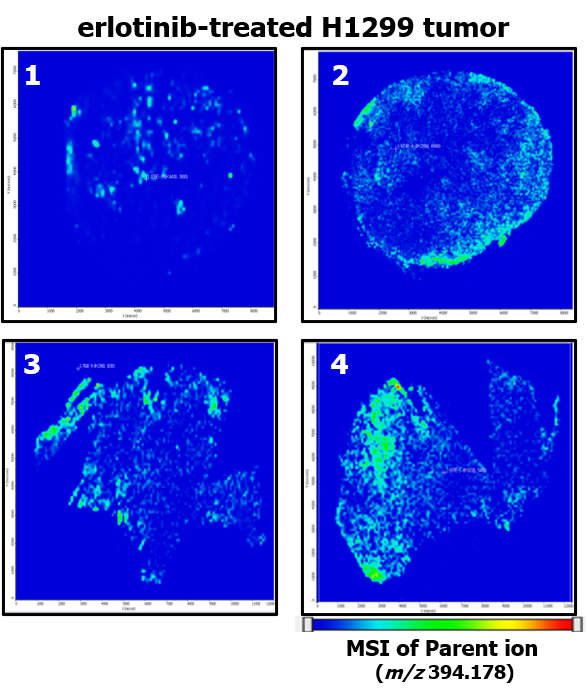


**Figure S7. The MALDI-MSI data of H1299 tumor tissue from H1299 xenograft mouse model.** Distribution of erlotinib in drug-treated tumor tissue by MALDI-MSI from H1299 xenograft mouse. The images show the distribution of the erlotinib signal (*m/z* 394.178). Signal intensity distributions of the specific *m/z* values are shown as an RGB gradient from blue (low) to red (high). Each image represents a tumor sample from different xenograft mouse.


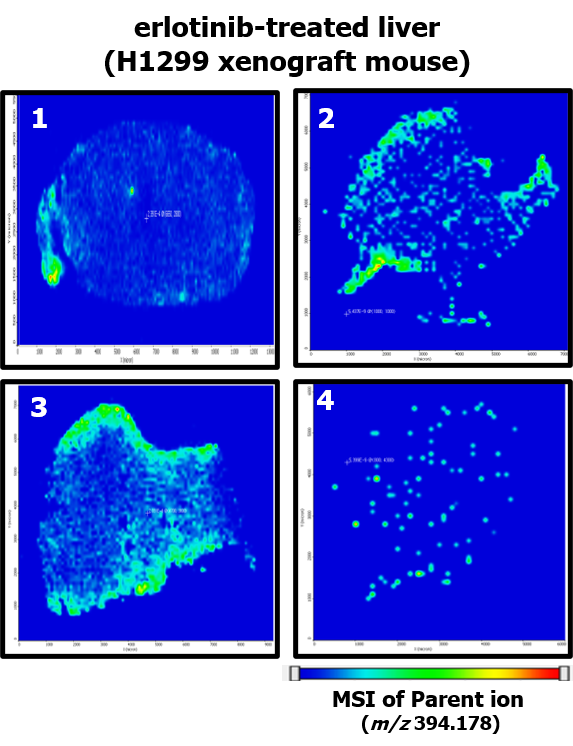


**Figure S8. The MALDI-MSI data of drug-treated liver tissue from H1299 xenograft mouse model.** Distribution of erlotinib in drug-treated liver tissue by MALDI-MSI from H1299 xenograft mouse. The images show the distribution of the erlotinib signal (*m/z* 394.178). Signal intensity distributions of the specific *m/z* values are shown as an RGB gradient from blue (low) to red (high). Each image represents a tumor sample from different xenograft mouse.


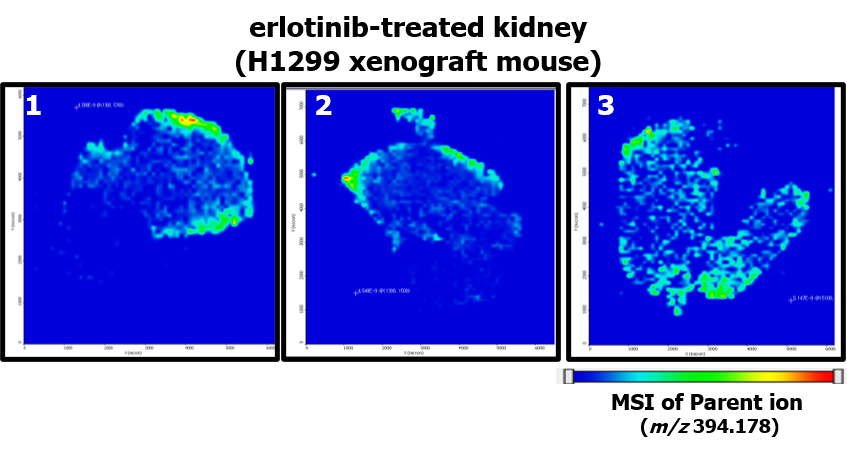


**Figure S9. The MALDI-MSI data of drug-treated kidney tissue from H1299 xenograft mouse model.** Distribution of erlotinib in drug-treated kidney tissue by MALDI-MSI from H1299 xenograft mouse. The images show the distribution of the erlotinib signal (*m/z* 394.178). Signal intensity distributions of the specific *m/z* values are shown as an RGB gradient from blue (low) to red (high). Each image represents a tumor sample from different xenograft mouse.


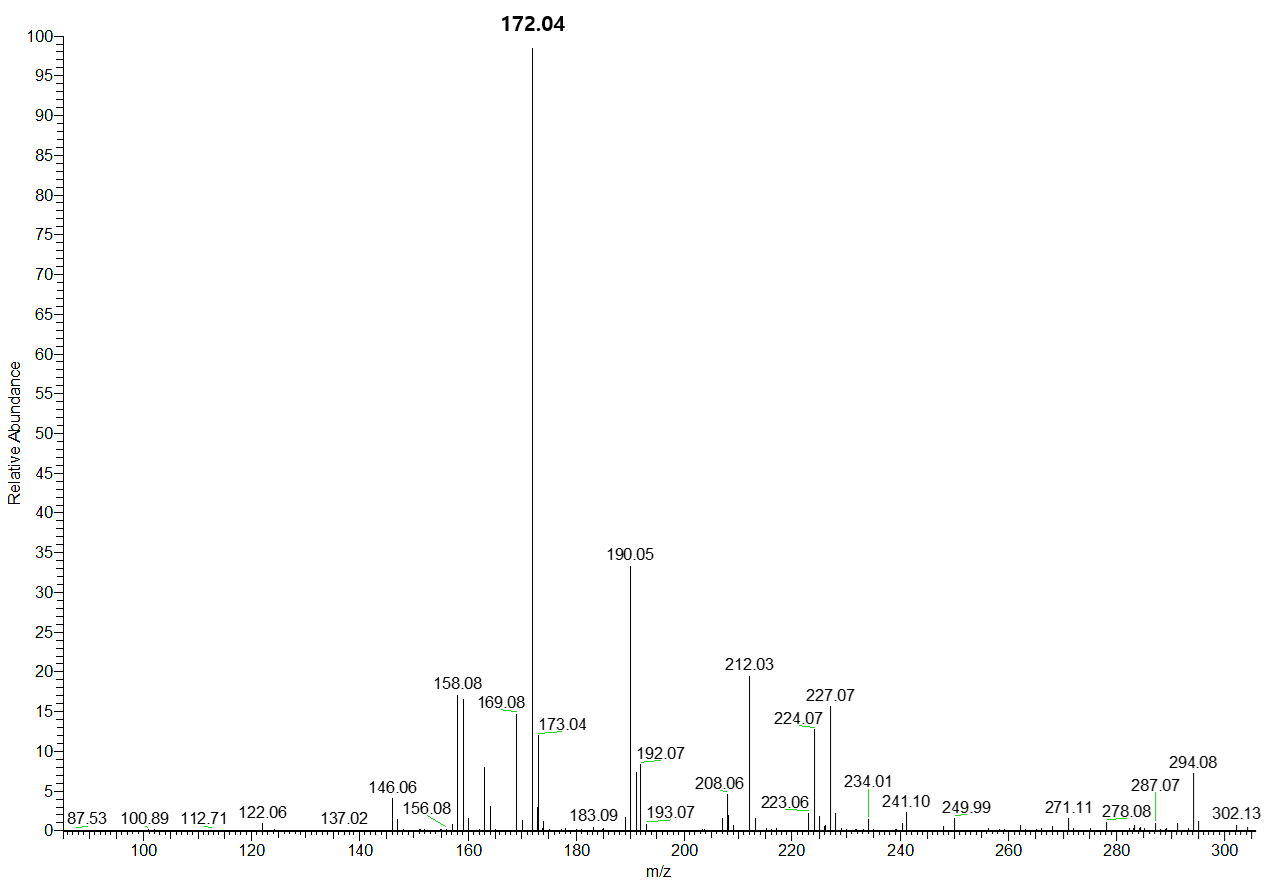


**Figure S10. Ionization** **α-CHCA.** Full mass spectrum of α-CHCA (*m/z* 172.04, [MH-H_2_O]^+^).[^16^](#_ENREF_16) A value of *m/z* in this experiment was confirmed in MALDI plate. This result was used to apply to the MALDI-MSI data of Figure S11 to lead to visualize the distribution of α-CHCA in tumor tissue samples.


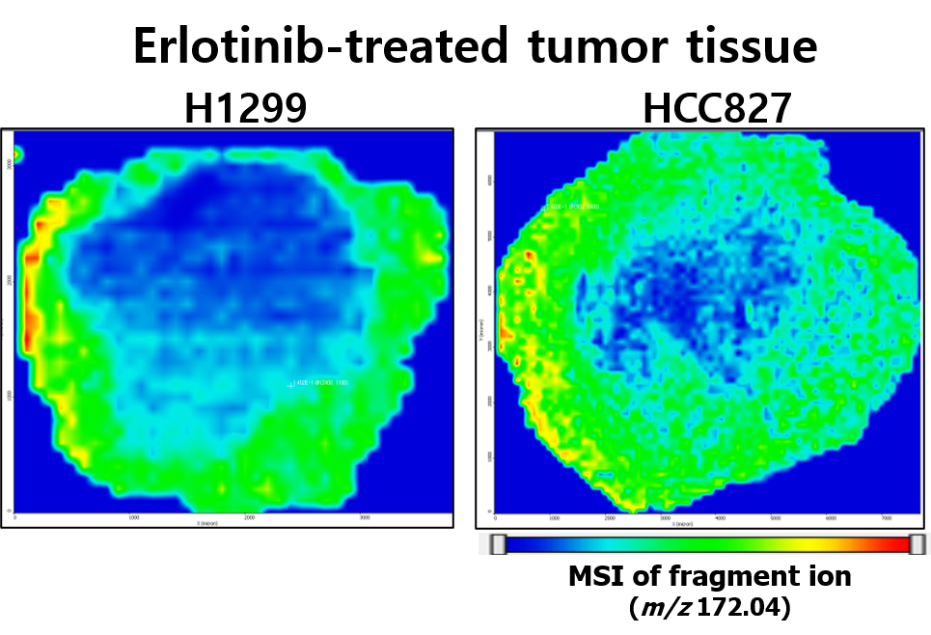


**Figure S11. MALDI-MSI data of α-CHCA from tumor in mouse xenograft tissue.** Distribution levels of α-CHCA in Erlotinib-treated mouse xenografts were analyzed with MALDI-MSI to exhibit the reproducibility of other MSI data. The images show the signal intensity distribution at *m/z* 172.04. The intensity of specific *m/z* values are shown as an RGB gradient from blue (low) to red (high).


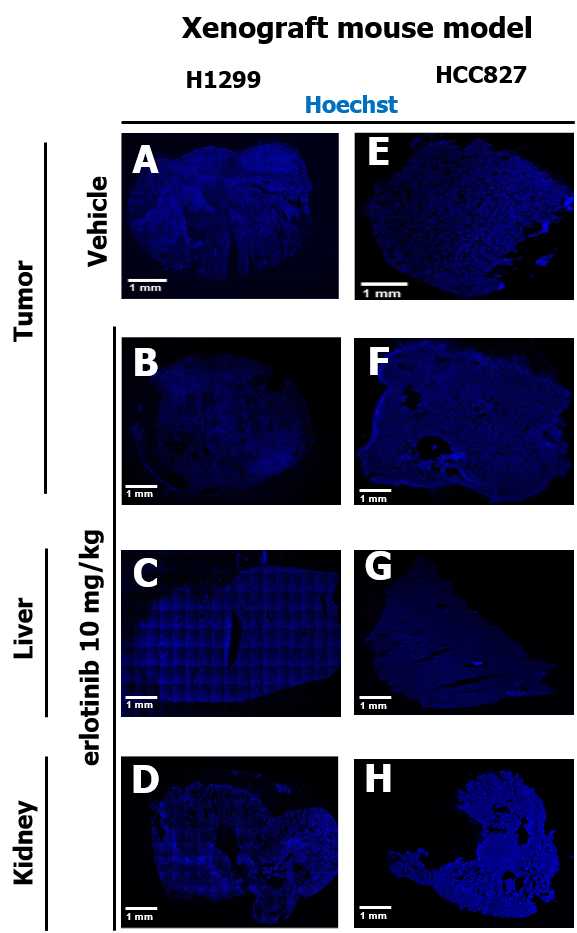


**Figure S12. The distribution of nucleus in mouse xenograft model with H1299 and HCC827 cells.** (A-H) Distribution of nucleus in vehicle or erlotinib-treated tissue (tumor, liver, and kidney) visualized with immuno-fluorescence (IF). The nucleus is visualized with Hoechst 33342 (blue). The xenograft mice were treated with either vehicle (N = 4) or erlotinib (N = 5) (10 mg/kg) for analyzing erlotinib distribution.


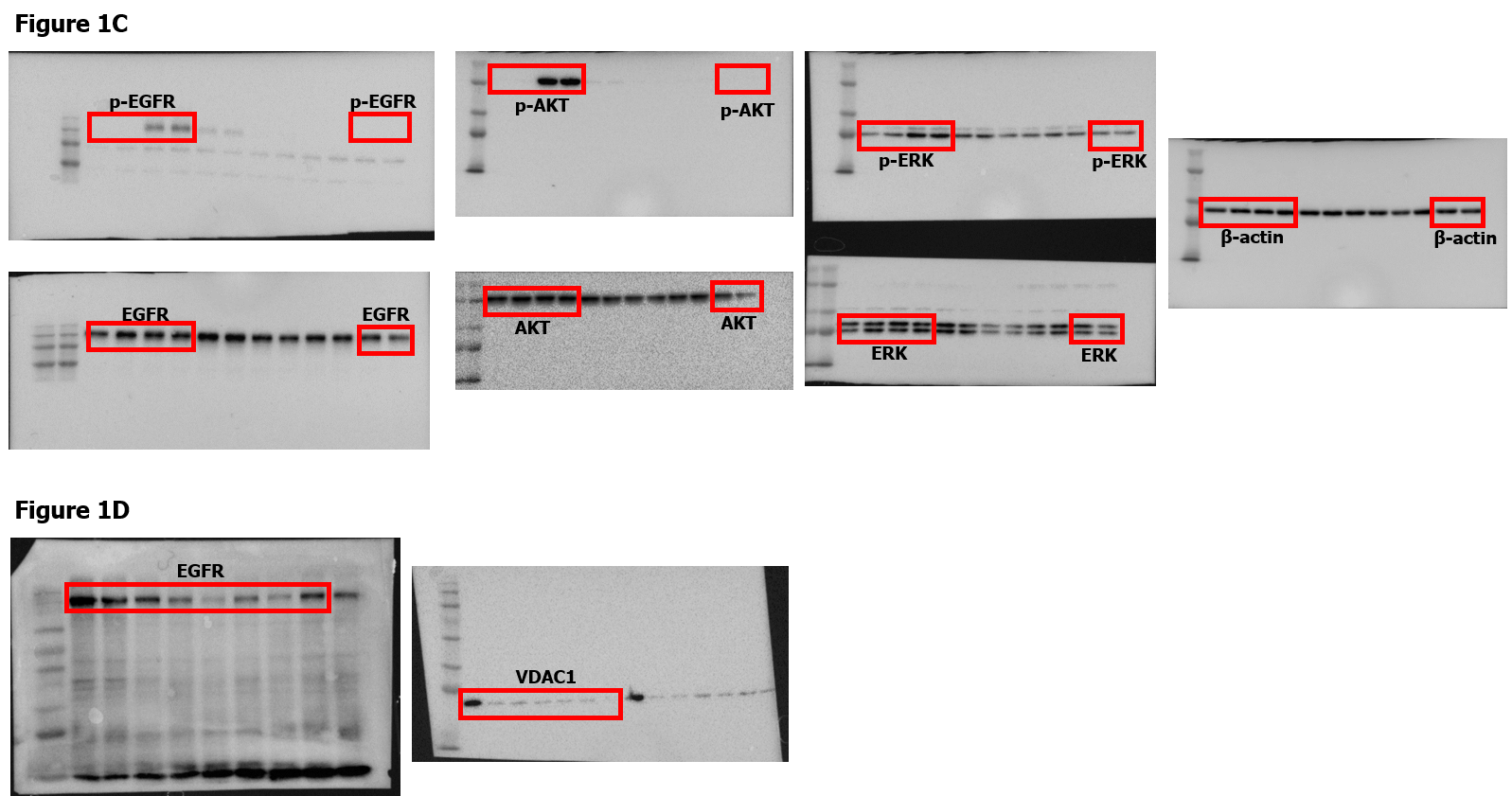


**Figure S13. The full blots for all of western blot used in main figures.**

*Tae Young Kim^1^,*

*Boram Lee^2^,*

*Yonghyo Kim^2^,*

*Yutaka Sugihara^2^,*

*Melinda Rezeli^2^,*

*A. Marcell Szasz^2, 3^,*

*Balazs Dome^3,4^,*

*Gyorgy Marko-Varga^2^,*

*Ho Jeong Kwon^1,*^*

*^1^Chemical Genomics Global Research Lab, Department of Biotechnology, College of Life Science & Biotechnology, Yonsei University, Seoul 03722, Korea*

*^2^ Division of Clinical Protein Science & Imaging, Dept. of Clinical Sciences (Lund) and Dept. of Biomedical Engineering, Lund University, SE-221 84, Lund, Sweden*

*^3^ Hungarian Academy of Sciences, 1117 Budapest, Hungary; Department of Thoracic Surgery, National Institute of Oncology and Semmelweis University, Budapest, Hungary*

*^4^ Division of Thoracic Surgery, Department of Surgery, Medical University of Vienna, Vienna, Austria*

***Correspondence:** Ho Jeong Kwon

Chemical Genomics Global Research Lab, Department of Biotechnology, College of Life Science & Biotechnology, Yonsei University, Seoul 03722, Korea.

E-mail:[kwonhj@yonsei.ac.kr](mailto:kwonhj@yonsei.ac.kr)

**Reference**

1. Chang, J., Kim, Y., Kwon, H. J. Advances in identification and validation of protein targets of natural products without chemical modification. *Nat Prod Rep* 2016;33 (5):719-730.

2. Hwang, H.-Y., Kim, T. Y., Szász, M. A., Dome, B., *et al.* Profiling the protein targets of unmodified bio-active molecules with drug affinity responsive target stability and liquid chromatography/tandem mass spectrometry. *Proteomics* doi:10.1002/pmic.201900325:1900325.

3. Inno, A., Di Noia, V., Martini, M., D'Argento, E., *et al*. Erlotinib for patients with EGFR wild-type metastatic NSCLC: a retrospective biomarkers analysis. *Pathol Oncol Res* 2019;25 (2):513-520.

4. Carey, K. D., Garton, A. J., Romero, M. S., Kahler, J., *et al.* Kinetic analysis of epidermal growth factor receptor somatic mutant proteins shows increased sensitivity to the epidermal growth factor receptor tyrosine kinase inhibitor, erlotinib. *Cancer Research* 2006;66 (16):8163-8171.

5. Walter, A. O., Sjin, R. T., Haringsma, H. J., Ohashi, K., *et al.* Discovery of a mutant-selective covalent inhibitor of EGFR that overcomes T790M-mediated resistance in NSCLC. *Cancer Discov* 2013;3 (12):1404-1415.

6. Wang, T., Zhang, J., Wang, S., Sun, X., *et al.* The exon 19-deleted EGFR undergoes ubiquitylation-mediated endocytic degradation via dynamin activity-dependent and -independent mechanisms. *Cell Commun Signal* 2018;16 (1):40.

7. Sawai, A., Chandarlapaty, S., Greulich, H., Gonen, M., *et al.* Inhibition of Hsp90 down-regulates mutant epidermal growth factor receptor (EGFR) expression and sensitizes EGFR mutant tumors to paclitaxel. *Cancer Res* 2008;68 (2):589-596.

8. Gao, S. P., Chang, Q., Mao, N., Daly, L. A., *et al.* JAK2 inhibition sensitizes resistant EGFR-mutant lung adenocarcinoma to tyrosine kinase inhibitors. *Sci Signal* 2016;9 (421):ra33.

9. Pinilla-Macua, I., Grassart, A., Duvvuri, U., Watkins, S. C., Sorkin, A. EGF receptor signaling, phosphorylation, ubiquitylation and endocytosis in tumors *in vivo*. *elife* 2017;6.

10. Al-Akhrass, H., Naves, T., Vincent, F., Magnaudeix, A., *et al*. Sortilin limits EGFR signaling by promoting its internalization in lung cancer. *Nat. Commun* 2017;8 (1).

11. Galetti, M., Petronini, P. G., Fumarola, C., Cretella, D., *et al*. Effect of ABCG2/BCRP Expression on Efflux and Uptake of Gefitinib in NSCLC Cell Lines. *PLoS One* 2015;10 (11):e0141795.

12. Schinkel, A. H., Jonker, J. W. Mammalian drug efflux transporters of the ATP binding cassette (ABC) family: an overview. *Adv Drug Deliv Rev* 2003;55 (1):3-29.

13. Bivona, T. G., Hieronymus, H., Parker, J., Chang, K., *et al.* FAS and NF-kappaB signalling modulate dependence of lung cancers on mutant EGFR. *Nature* 2011;471 (7339):523-526.

14. Naumov, G. N., Nilsson, M. B., Cascone, T., Briggs, A., *et al.* Combined vascular endothelial growth factor receptor and epidermal growth factor receptor (EGFR) blockade inhibits tumor growth in xenograft models of EGFR inhibitor resistance. *Clin Cancer Res* 2009;15 (10):3484-3494.

15. Mack, P. C., Holland, W. S., Burich, R. A., Sangha, R., *et al.* EGFR mutations detected in plasma are associated with patient outcomes in erlotinib plus docetaxel-treated non-small cell lung cancer. *J Thorac Oncol* 2009;4 (12):1466-1472.

16. Guo, Z., Zhang, Q., Zou, H., Guo, B., Ni, J. A method for the analysis of low-mass molecules by MALDI-TOF mass spectrometry. *Anal. Chem* 2002;74 (7):1637-1641.
